# Validation of “the patient evaluation of emotional care during hospitalisation” questionnaire in intensive care unit patients

**DOI:** 10.1371/journal.pone.0277172

**Published:** 2022-11-16

**Authors:** Alejandra Fernández Trujillo, Helena Vallverdú Cartié, Begoña Roman Maestre, Jorge Lema Vazquez, Julian Berrade Zubiri

**Affiliations:** 1 Intensive care unit, Consorci Corporació Sanitaria Parc Taulí, Sabadell, Spain; 2 Consorci Hospitalari de Vic, Vic, Spain; 3 Faculty of Pholosophy, University of Barcelona, Barcelona, Spain; 4 Parc Sanitari Sant Joan de Deu, Barcelona, Spain; Flinders University, AUSTRALIA

## Abstract

**Objective:**

To validate the “Patient Evaluation of Emotional Care During Hospitalization” (PEECH) questionnaire, which assesses hospitalised patients’ emotional experiences, in patients admitted to the intensive care unit (ICU).

**Interventions:**

Prospective study. The PEECH consists of three sections and four sub-scales: “level of security”, “level of knowing”, “level of personal value”, and “level of connection”. The questionnaire was completed by 253 hospitalised patients. Expert judgement was used to analyse the content validity and factor analysis was performed to confirm construct validity. Cronbach’s alpha was used to measure the internal consistency of the four sub-scales.

**Results:**

In the confirmatory factor analysis of the four sub-scales, the weights of all questions were found to be significant (>1). The internal consistency of the PEECH questionnaire was 0.86 (Cronbach’s alpha) and the homogeneity index was high (>0.50).

**Conclusion:**

The PEECH questionnaire is a valid and reliable tool to evaluate the perception of emotional care in ICU patients. The information gathered can help provide more comprehensive care for patients in the ICU and in other hospitalised patients.

## Background

For healthcare professionals and laypeople alike, ‘intensive care’ is intrinsically linked to a cold, highly technological environment, where patients are rendered invisible by the machines keeping them alive while their stricken loved ones wait anxiously, dreading bad news [1,2]. However, we are now witnessing what Heras has described as an “emotional awakening” [3]. This has been illustrated by the positive uptake of the Humanisation of Intensive Care Units project (H-ICU) [4], which has highlighted the need in healthcare for a shift in focus to address the emotional needs of critical care patients and their relatives. This paradigm shift has spilled over into other areas of medicine. In an article entitled ‘The Name of the Dog’ [5], Safder stressed the importance of human contact between doctors and patients and of doctors taking an interest in the person beyond the illness in order to improve the impact and efficiency of care.

The primary aim of an intensive care unit (ICU) is to provide life support and care. Humanising implies ethical responsibility, which, according to Noddings [6], entails reciprocity in forging a unique relationship with the patient. In the ICU, each relationship must be effectively coordinated with the rest of the team and the patient’s family members. This requires knowing more about the person than just their vital signs. Some patients may be unconscious for a time, but still undergo measurements of physiological constants, daily hygiene procedures, transfers for X-rays, etc. During this time, to ensure a genuinely reciprocal care relationship, hospital staff must ascertain the needs of the individual: their fears, hopes and joys when they begin to recover [7,8]. Viewed in this light it is clear that fully connecting to the emotional state of patients is vital to ensure a comprehensive and satisfactory care process.

ICU doctors are increasingly aware of the importance of emotional care to both patients and their families, and are setting more store in the process itself, rather than merely considering the outcomes. Indeed, patient and family satisfaction has become one of the key points in the “quality indicators in the critically ill” [9–12]. A growing number of publications have focused on the emotional needs of ICU patients and their relatives [13–15]. The importance of teaching communication skills to trainee residents and of preserving their capacity for compassion is becoming increasingly clear, as indicated in various studies [16,17]. In this sense, nurses have a head start over doctors, as they undergo far more comprehensive care training.

Although previous studies have found that patients and their families are broadly satisfied with healthcare provision in the ICU, there is still room for improvement in terms of care and emotional experience [11]. The Patient Evaluation of Emotional Care during Hospitalisation (PEECH) questionnaire explores this aspect of care and has been validated in hospitalised patients, including Spanish-speaking patients (using a Spanish-language version) [18–20]. The objective of this study was to validate the PEECH in patients admitted to the ICU.

## Materials and methods

### Study approach

A prospective study was carried out at the Parc Sanitari Sant Joan de Déu General Hospital in Barcelona, Spain, from September 2017 to May 2019.

Parc Sanitari Sant Joan de Déu is a public hospital with 200 beds that provides surgical and medical services for acute patients. The ICU was a 6-bed multi-purpose unit for critical and intermediate care patients where intensive care staff treat medical and surgical patients.

### Description of the questionnaire

The PEECH was selected from several possible questionnaires because it contains the most questions on the relationship between patients and staff. This was important: as this paper is part of a broader study on ethics in the ICU, we sought to center our analysis on care rather than satisfaction alone.

The questionnaire includes 35 questions split into three sections. The first section on healthcare staff contains 18 quantitative questions with the following possible answers: “all”, “most”, “some”, or “none”. The 4 questions in the second section could be answered with “always”, “usually”, “sometimes or “never”. The 13 questions on personal characteristics were yes/no answers, with a space for comments.

The questions in the three aforementioned sections comprise four sub-scales: [21].

- Level of security: vulnerability due to illness and dependence on others has a negative influence on the patient’s emotional experience. Patients feel secure when they perceive that staff are competent, they can establish relationships with them, and staff are available.
- Level of knowing: being informed about what is happening and what their healthcare process will entail improves patients’’ experience.
- Level of personal value: patients feel more valued if they have adequate communications with healthcare staff.
- Level of connection: patients feel continuity of contact and therefore have a better experience when there is continuity of contact with staff and patients can establish amiable relationships with staff.

### Patient recruitment

The inclusion criteria were as follows: ICU hospitalization for >24 hours, without prior general hospitalization; age >18 years; adequate state of consciousness of at least 24 hours; and ability to complete the questionnaire.

ICU patients were selected after consulting ICU staff about patients who fulfilled the inclusion criteria. After selection, participants were given an envelope containing the informed consent document and the questionnaire. All patients were informed that participation was voluntary. Completed documents were submitted by participants to hospital staff, who placed them in a box provided by the researchers.

Between September 2017 and May 2019, 844 patients were admitted to the ICU. From those who participated in the study 253 correctly completed questionnaires were obtained. No records were kept of patients who refused to participate in the study or of the number of incomplete questionnaires that were discarded.

### Ethical and legal considerations

The study protocol was approved by the Ethical Committee for Clinical Research of Parc Sanitari Sant Joan de Déu Hospital, on November 12, 2015 (internal code: PIC-108-15).

Because the surveys were not anonymous, informed consent was obtained from all participants. Data were processed according to applicable regulations.

### Validation

For validation, we calculated a necessary sample size of >100 questionnaires. This number was based on previous validation studies, including the validation of the Spanish-language version of the PEECH [20] that was used in the present study.

The Kaiser-Meyer-Olkin (KMO) measure of sampling adequacy provided a value of 0.864 (>0.5), while the Bartlett test value was <0.001, providing confirmatory factor analysis (CFA) of the construct validity. The aim of that analysis was twofold: to determine whether if the questions were appropriately categorised in the four sub-scales defined using the original version; and to ascertain whether these sub-scales adequately explain the results of the 22 questions in the first part of the questionnaire.

The reliability analysis was performed based on internal consistency measures. Cronbach’s alpha (values ≥0.7 indicate acceptable reliability) was calculated. We also calculated the homogeneity index: questions for which values <2.0 were obtained that questions that scored under 0.20 must be removed [21].

As in previous PEECH validation studies, mean scores were classified in two categories: low (≤2) and high (>2). The former were taken as an indicator of a need for emotional care. The percentage of responses to each was determined for each sub-scale. Chi-squared tests were performed to examine the relevance of patient characteristics for the lowest scores (worst level of sub-scale) and to identify specific patient profile patterns.

## Results

### Patient demographics

Between September 2017 and May 2019, 253 questionnaires were collected. The mean (standard deviation, SD) age of ICU patients was 65.8 (SD 16) years. The patient population comprised 154 (60.9%) women and 96 (37.9%) men. Of these, 9.9% had some difficulty in communicating (speech difficulty, 25%; hearing difficulty, 64.3%; visual difficulty, 10.7%). In addition, 68.8% of patients presented no diseases or functional limitations other than the reason for their current admission. In response to questions on whether they presented illnesses or functional limitations, 30% of ICU patients answered affirmatively. “Tables 1 and 2”.

**Table 1:** Characteristics of patients admitted to the ICU (n = 253).

	n	%
**Age (years)**, media (DE)	65,.8 (16,.0)	
**Sex**		
Male	154	60,.9
Female	96	37,.9
Unknown	3	1,.2
**Area of residence**		
Metropolitan	235	92,.9
Rural	14	5,.5
Unknown	4	1,.6
**Difficulty communicating**		
No	225	88,.9
Yes	25	9,.9
Unknown	3	1,.2
**Type of difficulty communicating**		
Speech	7	25,.0
Hearing	18	64,.3
Visual	3	10,.7
**Other diseases or functional limitations**		
No	174	68,.8
Yes	75	29,.6
Unknown	4	1,.6
**Days hospitalised during this admission**, mean (SD)	3.4 (4.5)	
1–3 days	179	70.7
> 3 days	74	29.3
**More than 30 days in hospital in the last year** ^**1**^		
No	228	91.2
Yes	22	8.8

Data are presented as frequency (n) and percentage (%) unless otherwise indicated.

SD, standard deviation.

1 City or urban area.

**Table 2:** Other patient and admission characteristics according to whether admitted to ICU (questions 29–35).

	ICU	
	n	%
Patients	253	
Do you currently need other people to help you in your day-to-day activities in hospital (e.g.: washing, walking, eating)? No Yes	123 126	49.4 50.6
Do you have family or friends who can help you in hospital if needed? No Yes	21 228	8.4 91.6
Is this the first time you have been admitted to hospital? No Yes	210 42	83.3 16.7
Were you admitted this time because of a new illness or recent injury? No Yes	68 180	27.4 72.6
Were you admitted in the last 24 hours? No Yes	137 107	56.2 43.8
Have you been in hospital for more than 3 days? No Yes	86 164	34.4 65.6
Are you in a single room for isolation? No Yes	137 111	55.2 44.8

### Description of the sub-scales and questions

The four sub-scales comprise 22 questions: level of security (6 questions), level of knowing (3 questions), level of personal value (10 questions), and level of connection (3 questions) “Table 3”.

**Table 3:** Sub-scales of the PEECH questionnaire for ICU patients.

**Level of Security**
Q1. Nurses help
Q2. Nurses contact
Q3. Staff competent
Q6. Staff respond
Q19. Overall secure
Q20. Overall supported
**Level of knowledge**
Q8. Nurses explains
Q9. Doctors explain
Q21. Overall iInformed
**Personal value level **
Q10. Staff eye contact
Q11. Staff distance
Q12. Staff voice
Q13. Staff caring
Q14. Staff encouraging
Q15. Staff listen
Q16. Staff expectations
Q17. Staff facial expression
Q18. Staff conversation
Q22. Overall valued
**Connection level**
Q4. Staff as people
Q5. Me as a person
Q7. 24-hour staff

Almost all patients answered all questions. “Table 4” shows the frequency distribution of responses to the various questions. Analysis of the non-response pattern shows that for all sub-scales the response rate was >96%.

**Table 4:** Frequency of answers to questions.

Sub-scale and question											N		% of answers		None	Some	*Most*			All	Never	Sometimes	Mostly	Always
**Level of Security **																								
Q1. Nurses help											252		99.6		1 (0.4%)	9 (3.6%)	25 (9.9%)			217 (86,1%)				
Q2. Nurses contact											251		99,2		2 (0,8%)	7 (2,8%)	26 (10,3%)			216 (86.1%)				
Q3. Staff competent											251		99,2		0 (0.0%)	2 (0,8%)	32 (12.7%)			217 (86,5%)				
Q6. Staff respond											252		99,6		1 (0,4%)	3 (1,2%)	14 (5,5%)			234 (92,9%)				
Q19 Overall secure											253		100,0								0 (0,0%)	9 (3,6%)	38 (15,0%)	206 (81,4%)
Q20. Overall supported											252		99,6								1 (0,4%)	1 (0,4%)	36 (14,3%)	214 (84,9%)
**Level of knowing**																					9	55	58	33
Q8. Nurses explain											250		98,8		19 (7,6%)	28 (11,2%)	46 (18,4%)			157 (62,8%)				
Q9. Doctors explain											249		98,4		6 (2,4%)	6 (2,4%)	24 (9,6%)			213 (85,6%)				
Q21. Overall informed											253		100,0								2 (0,8%)	6 (2,4%)	39 (15,4%)	206 (81,4%)
**Level of Personal value **																								
Q10. Staff eye contact											246		97,2		2 (0,8%)	9 (3,7%)	27 (11,0%)			208 (84,5%)				
Q11 Staff distance											251		99,2		2 (0.8%)	6 (2.4%)	14 (5,6%)			229 (91,2%)				
Q12. Staff voice											251		99,2		1 (0,4%)	1 (0,4%)	11 (4,4%)			238 (94,8%)				
Q13. Staff caring											252		99,6		0 (0,0%)	1 (0,4%)	17 (6,7%)			234 (92,9%)				
Q14. Staff encouraging											244		96,4		3 (1,2%)	2 (0,8%)	36 (14,8%)			203 (83,2%)				
Q15. Staff listen											250		98,8		1 (0,4%)	6 (2,4%)	26 (10,4%)			217 (86,8%)				
Q16. Staff expectations											252		99,6		2 (0,8%)	2 (0,8%)	29 (11,5%)			219 (86,9%)				
Q17. Staff facial expression											249		98,4		5 (2,0%)	2 (0,8%)	29 (11,7%)			213 (85,5%)				
Q18. Staff conversation											246		97,2		24 (9,8%)	47 (19,1%)	50 (20,3%)			125 (50,8%)				
Q22. Overall valued											253		100,0								0 (0,0%)	2 (0,8%)	23 (9,1%)	227 (89,7%)
**Level of Connection**																								
Q4. Staff as people			249			98,4			8 (3,2%)					68 (27,3%)	84 (33,7%)	89 (35,7%)								
Q5. Me as person			251			99,2			14 (5,6%)					60 (23,9%)	73 (29,1%)	104 (41,4%)								
Q7. 24-hour staff			243			96,1			130 (53,5%)					46 (18,9%)	22 (9,1%)	45 (18,5%)								

### Psychometric properties

The evaluation of the psychometric properties of the PEECH questionnaire was divided into a reliability analysis and a validity analysis.

### Validity analysis

The confirmatory factor analysis (CFA) confirmed four extract factors corresponding to the four sub-scales of the questionnaire: eigenvalues >1 were obtained for the four first factors, which explained 70% of the total variance of the data.

### Reliability analysis

The reliability analysis was performed based on internal consistency measures.

According to Cronbach’s alpha, the internal consistency of the PEECH questionnaire for patients admitted to the ICU was 0.86 (i.e. good). Analysis of each sub-scale revealed satisfactory internal consistency (> or very close to 0.7). Two sub-scales had a Cronbach’s alpha values were slightly below 0.7 (0.68 for level of knowing and 0.67 for level of connection) “Table 5”.

**Table 5:** Internal consistency of the PEECH questionnaire and each of its sub-scales.

Sub-scale	N° of Questions	Cronbach’s alpha
Level of security	6	0,72
Level of knowledge	3	0,68
Level of personal value	10	0,79
Level of connection	3	0,67
**PEECH**	**22**	**0,86**

A high homogeneity index (>0.50) was obtained for all questions in the questionnaire. None had a homogeneity index lower than 0.2, which would have required deletion of the question. “Table 6”.

**Table 6:** Homogeneity index of each question and internal consistency of the PEECH questionnaire after elimination of the question.

Item	Homogeneity index	Cronbach’s alpha after removal of item
**Level of Security **		
Q1. Nurses help	0,705	0,85
Q2. Nurses contact	0,731	0,85
Q3. Staff competent	0,569	0,85
Q6. Staff respond	0,713	0,85
Q19. Overall secure	0,572	0,85
Q20. Overall supported	0,607	0,85
**Level of Knowledge **		
Q8. Nurses explain	0,862	0,85
Q9. Doctors explain	0,772	0,85
Q21. Overall informed	0,755	0,85
**Level of Personal value **		
Q10. Staff eye contact	0,611	0,85
Q11. Staff distance	0,588	0,85
Q12. Staff voice	0,501	0,86
Q13. Staff caring	0,583	0,86
Q14. Staff encouraging	0,627	0,85
Q15. Staff listen	0,717	0,85
Q16. Staff expectations	0,788	0,85
Q17. Staff facial expression	0,603	0,85
Q18. Staff conversation	0,668	0,85
Q22. Overall valued	0,641	0,85
**Level of Conection **		
Q4. Staff as people	0,785	0,85
Q5. Me as person	0,774	0,85
Q7. 24 hours staff	0,785	0,87

The total score calculated in each sub-scale indicated a low or moderate and statistically significant correlation between all sub-scales. The most significant correlation was between level of security and level of personal worth (0.582). The total score calculated from the PEECH questionnaire was significantly correlated with all sub-scales, with a good correlation for the level of knowing (0.673) and level of security (0.702) sub-scales and a very good correlation for the level of personal value (0.818) and level of connection (0.823) sub-scales “Table 7”.

**Table 7:** Correlations between sub-scales (n = 253).

Sub-scales	Sub-scales			
	Level of security	Level of knowledge	Level of personal value	Level of connection
Level of security	1			
Level of knowledge	0,468*	1		
Level of personal value	0,582*	0,458*	1	
Level of connection	0,412*	0,400*	0,531*	1
**PEECH (22)**	**0,702** *	**0,673** *	**0,818** *	**0,823** *

* Statistically significant Spearman correlation coefficient (p <0.05); SD, standard deviation.

### Patient profile

Scores were classified as ≤2 or >2. The percentage of patients with lower scores for perception of care, mean scores ≤2 were considered typical of patients potentially in need of additional care, in agreement with the validation of the original questionnaire [18,21]. Scores >2 were obtained for 96.8% of ICU patients for the sub-scale level of security, 81.5% for level of knowing, 96.9% for level of personal value, and 23.8% for level of connection. Overall mean scores >2 were obtained for level of security (2.83±0.29), level of knowing (2.64±0.56), and level of personal value (2.79±0.31), and <2 for level of connection (1.66±0.78) “Table 8”.

**Table 8:** Frequency and percentage of patients in each sub-scale split into two categories (low and high scores).

	ICU	
Mean scores	n	%
**Level of security**		
Lowest scores ≤2	8	3.2
Highest scores >2 or = 3	241	96.8
Total	249	
**Level of knowing**		
Lowest scores ≤2	46	18.5
Highest scores >2 or = 3	202	81.5
Total	248	
**Level of personal value**		
Lowest scores ≤2	7	3.1
Highest scores >2 or = 3	218	96.9
Total	225	
**Level of connection**		
Lowest scores ≤2	183	76.2
Highest scores >2 or = 3	57	23.8
Total	240	

3 is the best possible answer and 0 is the worst.

## Discussion

The present findings demonstrate that the PEECH questionnaire prepared by Williams [18], the Spanish language version of which was validated in 2019 [20], is valid for use in ICU patients.

The objective of ICU care is to provide patients with high quality health care that is adjusted to their needs, as safe as possible, and is adequate, sustainable, ethical, and respectful of the patient’s autonomy. By exploring this dimension, we believe that we can determine whether a patient *feels* cared for, as well as *knowing* they are cared for.

When patients are admitted to ICU, they experience stress and a palpable loss of independence [22]. Numerous studies have examined ICU patient and family satisfaction [11–13]. Satisfaction is one of the quality criteria considered by the Spanish Society of Intensive Medicine Critical Medicine and Coronary Units (SEMICYUC) [9,10]. However, emotional dimensions may be additional to trust in the technical skills of hospital staff. This is one of the reasons why ICU can be considered a “branch of hell” [1], while at the same time scoring well in satisfaction surveys [22,23].

In the original version of the PEECH designed by Williams and Kristjanson [18], the questionnaire had three sub-scales: security level, knowledge level and personal value. In the factorial analysis phase, a fourth sub-scale emerged: connection level. This encompassed questions originally assigned to the security level sub-scale that explored the relationship between staff and patients. Subsequently, to assess its validity, Williams et al distributed the questionnaire to a larger number of patients, this time considering four distinct sub-scales. Their results confirmed the validity of the questionnaire to measure reciprocity in the relationship between the patient and hospital staff. This item is of particular interest when assessing how care is perceived, as it reflects the value of the patient as a person, not just as a patient. It allows for a better relationship, for the patient’s opinion to be valued, and for greater patient participation in the care process. Robert et al. [19] performed an internal validation of the same questionnaire in UK hospitals, and their analysis confirmed the presence of the fourth sub-scale. The factorial analysis in the Spanish-language version of the PEECH confirmed the presence of the connection level sub-scale. This sub-scale is also confirmed in the validation of the Spanish version of the questionnaire [20] The questionnaire was initially validated in a population of 132 patients, as in this study. A subsequent study evaluated the experience of emotional care in a hospital setting in a larger number of patients, and confirmed the utility of the PEECH. In the UK study, 423 patients were recruited. In the present study, the main objective was to validate the Spanish translation of the PEECH, for which an N >100 was calculated. Based on availability, it was decided to recruit a larger number of patients. The results obtained were similar to those reported by Williams et al [21] and by the study that validated the Spanish version of the questionnaire in hospitalized patients.

The overall mean scores were >2 for the sub-scales level of security, level of knowing, and level of personal value. A higher percentage of patients had a lower score for the level of connection sub-scale (mean score, 1.66). These findings are in agreement with those of Williams et al. and the results obtained during validation of the Spanish-language version of the PEECH [20,21]. These scores are interpreted as indicators of a need to address this area. This sub-scale was not included in the original questionnaire [18] and was incorporated after the initial analysis, comprising three questions originally assigned to the level of security sub-scale corresponding to the relationship between staff and patients. In Williams’ [21] second study, in which this sub-scale was included, the mean score was also <2. Williams attributed that result to a lack of reciprocity between staff and patients. However, analysis of the scores for the three questions that make this sub-scale reveals that one question exerts a greater statistical influence: “I had previously met the nursing staff that I have seen during the past 24 hours”. Of the four response options, most ICU patients replied “none”. By contrast, this option accounted for less than 12% of the answers to all other questions. Level of connection with staff is partly measured based on the continuity of healthcare given by the same staff members. In an acute care hospital with limited average stays (particularly in the ICU), continuity of contact is difficult due to shift systems. Nonetheless, this low score should be taken into account.

## Conclusion

The findings reported here and in previous validation studies indicate that the PEECH questionnaire is a valid, reliable, and useful instrument with which to evaluate the perception of emotional care in patients admitted to the ICU.

## Relevance for clinical practice

In the era of humanization of the ICU, the PEECH could be applied in daily clinical practice to identify patients with special needs, thereby helping to improve, and serving as an indicator of, the quality of care provided.

## Limitations

A limitation of the present study is that we did not record data on patients who refused to participate, nor could their characteristics be studied. Furthermore, the fact the ICU in question contained only six beds and has a particular interest in humanisation, providing specific staff training in this area, may have contributed to the high scores obtained. Further studies in larger ICUs with more staff will be necessary to corroborate the present findings.
